# Response Regulator CD1688 Is a Negative Modulator of Sporulation in Clostridioides difficile

**DOI:** 10.1128/jb.00130-22

**Published:** 2022-07-19

**Authors:** Megan L. Kempher, Savannah C. Morris, Tyler M. Shadid, Smita K. Menon, Jimmy D. Ballard, Ann H. West

**Affiliations:** a University of Oklahoma grid.266900.bgrid.266902.9 , Department of Chemistry and Biochemistry, Norman, Oklahoma, USA; b University of Oklahoma grid.266900.bgrid.266902.9 Health Sciences Center, Department of Microbiology and Immunology, Oklahoma City, Oklahoma, USA; Ohio State University

**Keywords:** *Clostridioides difficile*, response regulator, sporulation, two-component regulatory systems

## Abstract

Two-component signal transduction systems (TCSs), consisting of a sensor histidine kinase (HK) and a response regulator (RR), sense environmental stimuli and then modulate cellular responses, typically through changes in gene expression. Our previous work identified the DNA binding motif of CD1586, an RR implicated in Clostridioides difficile strain R20291 sporulation. To determine the role of this RR in the sporulation pathway in C. difficile, we generated a deletion strain of *cd1688* in the historical 630 strain, the homolog of *cd1586*. The C. difficile Δcd1688 strain exhibited a hypersporulation phenotype, suggesting that CD1688 negatively regulates sporulation. Complementation of the C. difficile Δcd1688 strain restored sporulation. In contrast, a nonphosphorylatable copy of *cd1688* did not restore sporulation to wild-type (WT) levels, indicating that CD1688 must be phosphorylated to properly modulate sporulation. Expression of the master regulator *spo0A*, the sporulation-specific sigma factors *sigF*, *sigE*, *sigG*, and *sigK*, and a signaling protein encoded by *spoIIR* was increased in the C. difficile Δcd1688 strain compared to WT. In line with the increased *spoIIR* expression, we detected an increase in mature SigE at an earlier time point, which arises from SpoIIR-mediated processing of pro-SigE. Taken together, our data suggest that CD1688 is a novel negative modulator of sporulation in C. difficile and contributes to mediating progression through the spore developmental pathway. These results add to our growing understanding of the complex regulatory events involved in C. difficile sporulation, insight that could be exploited for novel therapeutic development.

**IMPORTANCE** Clostridioides difficile causes severe gastrointestinal illness and is a leading cause of nosocomial infections in the United States. This pathogen produces metabolically dormant spores that are the major vehicle of transmission between hosts. The sporulation pathway involves an intricate regulatory network that controls a succession of morphological changes necessary to produce spores. The environmental signals inducing the sporulation pathway are not well understood in C. difficile. This work identified a response regulator, CD1688, that, when deleted, led to a hypersporulation phenotype, indicating that it typically acts to repress sporulation. Improving our understanding of the regulatory mechanisms modulating sporulation in C. difficile could provide novel strategies to eliminate or reduce spore production, thus decreasing transmission and disease relapse.

## INTRODUCTION

Clostridioides difficile is a human enteropathogenic bacterium currently categorized as an urgent health care threat by the CDC due to its multidrug resistance and increasing infection rate (roughly 500,000 cases per year) (1). This bacterium is a spore-forming, obligate anaerobe that colonizes the lower gut, causing gastrointestinal symptoms ranging from diarrhea to toxic megacolon and even death (2, 3). C. difficile infections commonly follow antibiotic treatment, which disrupts the protective gut microbiota, with recurring infections happening in 15 to 35% of cases (4). Unlike vegetative cells, spores can survive aerobic conditions and are highly resistant to chemical and physical cleaning procedures, making them the primary mode of transmission between hosts (5–9).

The overall steps of spore formation involve an intricate developmental pathway that is generally conserved in members of the *Firmicutes* phylum. The first step of sporulation is asymmetric division where the cell produces a larger mother cell and a smaller forespore. Sporulation initiation is controlled by the master regulator stage 0 sporulation protein A (Spo0A) (3, 9–12), which must be phosphorylated for the pathway to be activated (10, 12–14). As a transcriptional activator, phosphorylated Spo0A (Spo0A~P) binds DNA, initiating the expression of genes necessary for the early stages of sporulation, including the cell type-specific sigma factors *sigE* and *sigF* (9, 13, 15, 16). Inactivation of Spo0A in C. difficile results in an asporogenic phenotype similar to what is observed in Bacillus subtilis (10, 17). The signal transduction pathway that controls sporulation initiation was initially and extensively studied in B. subtilis (15, 18, 19). However, several recent studies have revealed many key differences in the sporulation pathway of C. difficile. In B. subtilis, Spo0A phosphorylation occurs via a multistep phosphorelay system consisting of multiple sensor kinases and phosphotransfer proteins (18). In C. difficile, phosphorylation of Spo0A is predicted to be modulated by several orphan histidine kinases (CD1492, CD1579, CD1942, and CD2492) (10, 17). CD1492 and CD2492 repress sporulation initiation by acting as phosphatases toward phosphorylated Spo0A~P (20, 21). CD1579 has been shown to phosphorylate Spo0A *in vitro* (10), although recent studies indicate that it may also repress sporulation (21, 22).

While the specific signals initiating Spo0A phosphorylation in C. difficile remain unknown, several studies have demonstrated a direct link between nutrient availability and sporulation initiation. Global regulators CodY, which responds to cellular levels of branched-chain amino acids (BCAAs) and GTP (23), and CcpA, which senses global carbon availability, repress sporulation when nutrients are abundant (24). These regulators also repress expression of the *sinRR′* locus, which encodes two transcriptional regulators that are known to influence transcription of *spo0A*, and genes encoding proteins that mediate motility and toxin production (25). Recently, transcriptional regulator CD2589 was shown to decrease the abundance of *spo0A* transcripts within the cell in response to available nutrients present in the environment (26).

Two-component signal transduction systems (TCSs) are composed of a histidine kinase (HK) and a response regulator (RR) that function as a unit to sense and respond to environmental signals. HKs detect an environmental signal (nutrients, stress, antibiotics, etc.) using a sensory domain and autophosphorylate at a conserved histidine residue (27). The RR is able to induce transfer of the phosphoryl group from this phosphorylated histidine to a conserved aspartate residue in the receiver domain of the RR (28), resulting in a conformational change that alters the biological activity of the RR. The effector domain of the RR determines the biological response, the most common of which is regulation of gene expression (29). Improving our understanding of gene regulation in C. difficile by TCSs could reveal new targets for therapeutic development, especially since these systems are absent in animals (30).

Previously, our lab used a bacterial one-hybrid system to determine the DNA motif that RR CD1586 recognizes and binds to in the hypervirulent C. difficile strain R20291 (CDR20291). These analyses identified multiple putative gene targets (31). These targets included genes encoding several ion transporters, ABC transporters, enzymes involved in proteolysis, and sporulation-related proteins. RR CD1586 had been previously implicated in a transposon mutagenesis screen as being important in sporulation (32). In C. difficile strain 630 (CD630), the homolog to CD1586 is CD1688 (100% amino acid identity), which has a cognate HK, CD1689. Here, we report the creation of a *cd1688* deletion strain in CD630, a more genetically tractable strain than R20291, that resulted in a hypersporulation phenotype. We further show that this increased sporulation was accompanied by increased expression of sporulation-specific genes and increased processing of sporulation-specific sigma factor SigE and was dependent on phosphorylation of CD1688. Deletion of *cd1688* had no effect on the expression of toxin genes or motility. Taken together, our results demonstrate that CD1688 functions to repress sporulation in C. difficile under certain environmental conditions.

## RESULTS

### Construction of a *cd1688* deletion strain.

**FIG 1:**
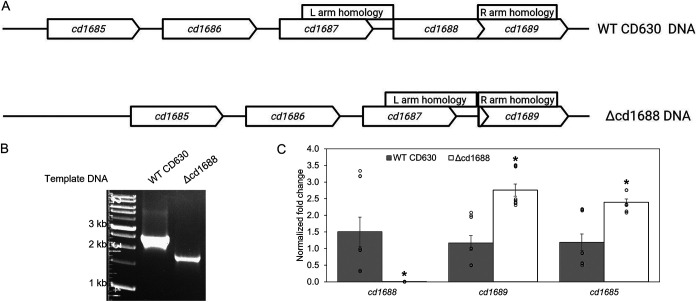
Deletion of the *cd1688* gene in C. difficile. (A) *cd1688* locus in C. difficile 630 wild-type (WT) and C. difficile Δcd1688 strains. Boxes indicate positions of homology arms used for construction of the deletion strain. (B) PCR confirmation of *cd1688* gene deletion in C. difficile Δcd1688. The *cd1688* gene deletion removed 661 bp. (C) Abundance of *cd1688*, *cd1689*, and *cd1685* in the C. difficile Δcd1688 strain relative to the WT strain measured via qRT-PCR. Three biological replicates of each strain were grown in BHIS to an OD_600_ of 1.0. *, *P* ≤ 0.05.

The genes encoding RR CD1688 (*cd1688*) and HK CD1689 (*cd1689*) are located in a putative five-gene operon downstream of a hypothetical protein (*cd1685*), a transcriptional regulator (*cd1686*), and a lipoprotein (*cd1687*) (Fig. 1A). To further interrogate the role of this TCS in C. difficile physiology, we deleted *cd1688* in wild-type (WT) CD630 using a CRISPR-Cas9 nickase gene editing system (here named C. difficile Δcd1688) (see Fig. S1 in the supplemental material) (33). To confirm the desired deletion was generated, genomic DNA (gDNA) was PCR amplified using primers that flanked the expected deletion (Table S2). As shown in Fig. 1B, C. difficile Δcd1688 was confirmed by an expected PCR product size decrease of 2,429 bp to 1,768 bp and further verified via Sanger sequencing. We next tested the growth of the C. difficile Δcd1688 strain compared to WT. The C. difficile Δcd1688 strain grew similarly to C. difficile WT in brain heart infusion medium supplemented with yeast extract (BHIS) (Fig. S2), indicating that the mutation did not cause any major growth defects under standard laboratory growth conditions.

### Expression of *cd1688* is likely controlled via autoregulation.

As shown in Fig. 1C, as expected, C. difficile Δcd1688 had no detectable *cd1688* transcripts, further confirming a clean deletion. To ensure the deletion of *cd1688* did not have any polar effects on the downstream gene, we measured the expression of *cd1689*, the gene encoding the cognate HK, using quantitative reverse transcription-PCR (qRT-PCR) analysis. Expression of *cd1689* was increased ~2.8-fold in C. difficile Δcd1688 compared to C. difficile WT during stationary growth in BHIS medium (Fig. 1C). Our previous work identified a putative binding motif of CD1688 upstream of *cd1685*, the predicted operon leader of this locus. Therefore, we also measured the expression of *cd1685* in the C. difficile Δcd1688 strain compared to the WT strain (Fig. 1C). Expression of *cd1685* was also increased in the deletion strain (~2.1-fold). Together, these data indicate that CD1688 likely autoregulates its own expression through regulation of the entire operon, as suggested by our previous work (31).

### The expression of several predicted gene targets of CD1688 was decreased in the C. difficile Δcd1688 strain.

**FIG 2:**
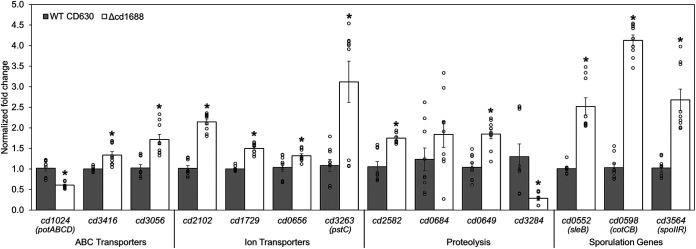
Transcript abundance of predicted gene targets of CD1688 in the C. difficile WT and Δcd1688 strains. The expression of genes involved in ABC/ion transport, proteolysis, and sporulation was measured from RNA samples isolated from three biological replicates during stationary-growth phase in BHIS medium via qRT-PCR (OD_600_, ~1.0). The expression of each gene in the Δcd1688 strain is measured relative to the expression in the WT strain in the same growth conditions. *, *P* ≤ 0.05.

We next sought to evaluate whether the expression of the predicted gene targets of CD1688, identified in our previous work (31), was affected in the C. difficile Δcd1688 strain versus the WT strain during growth in BHIS. The majority of the predicted targets can be categorized into one of the following functional groups: ABC/ion transport, proteolysis, or sporulation processes. Therefore, we tested the expression of the previously identified gene targets from these functional groups. All of these targets were significantly differentially expressed in the C. difficile *Δ*cd1688 strain, with the exception of *cd0684* (Fig. 2). The majority of targets had increased expression in the C. difficile Δcd1688 strain, indicating that CD1688 likely represses these targets in the growth conditions tested. Two targets, *cd1024* and *cd3284*, which encode the *potABCD* transporter and an HtrA-like protease, respectively, showed a decrease in expression in the C. difficile Δcd1688 strain.

### Deletion of *cd1688* increases sporulation efficiency in C. difficile 630 but has no effect on toxin production or motility.

**FIG 3:**
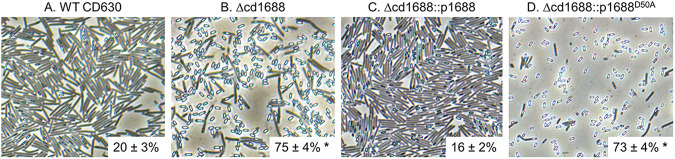
Sporulation efficiency of C. difficile WT versus Δcd1688 strains. Phase-contrast microscopy of C. difficile WT (A), Δcd1688 (B), Δcd1688::p1688 (C), and Δcd1688::p1688^D50A^ (D) grown on 70:30 sporulation agar supplemented with 0.1% xylose for 24 h. Inset numbers represent sporulation efficiency as measured by ethanol resistance assays. *, *P* ≤ 0.05 as determined by a one-way ANOVA followed by Dunnett’s multiple-comparison test compared to C. difficile WT630.

Since a previous transposon mutagenesis study indicated that *cd1688* was one of 798 genes likely to impact sporulation (32), we tested if deleting *cd1688* had any effect on the ability of C. difficile to produce spores. Phase-contrast microscopy was used to discriminate vegetative cells (phase dark) from spores (phase bright) after 24 h of growth on 70:30 sporulation plates. We saw a significant increase in the number of spores in the C. difficile Δcd1688 strain compared to the WT strain (Fig. 3). The sporulation efficiency was quantified by enumerating ethanol-resistant spores and vegetative cells after 24 h of growth on 70:30 sporulation plates. The C. difficile WT strain exhibited a sporulation efficiency of ~20%, which is similar to previously reported sporulation frequencies for WT CD630 (34) (Fig. 3A and Fig. S3). The C. difficile Δcd1688 strain had a sporulation efficiency of ~75%, which was ~3.75-fold higher than the WT strain (Fig. 3B and Fig. S3). These data strongly suggest that in the C. difficile WT strain, CD1688 acts to inhibit spore formation.

To confirm the hypersporulation phenotype was solely due to the deletion of *cd1688*, a complement strain was constructed by conjugating a vector that expresses the *cd1688* gene under a xylose-inducible promoter into the C. difficile Δcd1688 strain (here named C. difficile Δcd1688::p1688) (Fig. S4) (35). When 0.1% xylose was added to the growth medium, the sporulation efficiency of C. difficile Δcd1688::p1688 was restored to levels similar to C. difficile WT (~16%) (Fig. 3C and Fig. S3), further corroborating that the hypersporulation phenotype was due to the deletion of *cd1688*.

Since sporulation, toxin production, and motility are tightly linked via the activities of several global regulators in C. difficile (12, 24, 25, 36), we also tested if deleting *cd1688* had any effect on these processes. The C. difficile Δcd1688 strain did not show any significant changes compared to the WT strain in the expression of toxin genes *tcdA* or *tcdB* or the toxin-specific sigma factor *tcdR* (Fig. S5A), indicating that CD1688 does not appear to regulate toxin expression. We next tested motility of the C. difficile WT and Δcd1688 strains. Growth diameters were similar between the C. difficile WT and Δcd1688 strains (Fig. S5B), suggesting that CD1688 also does not affect cell motility.

### Deletion of *cd1688* affects expression of other global regulators.

**FIG 4:**
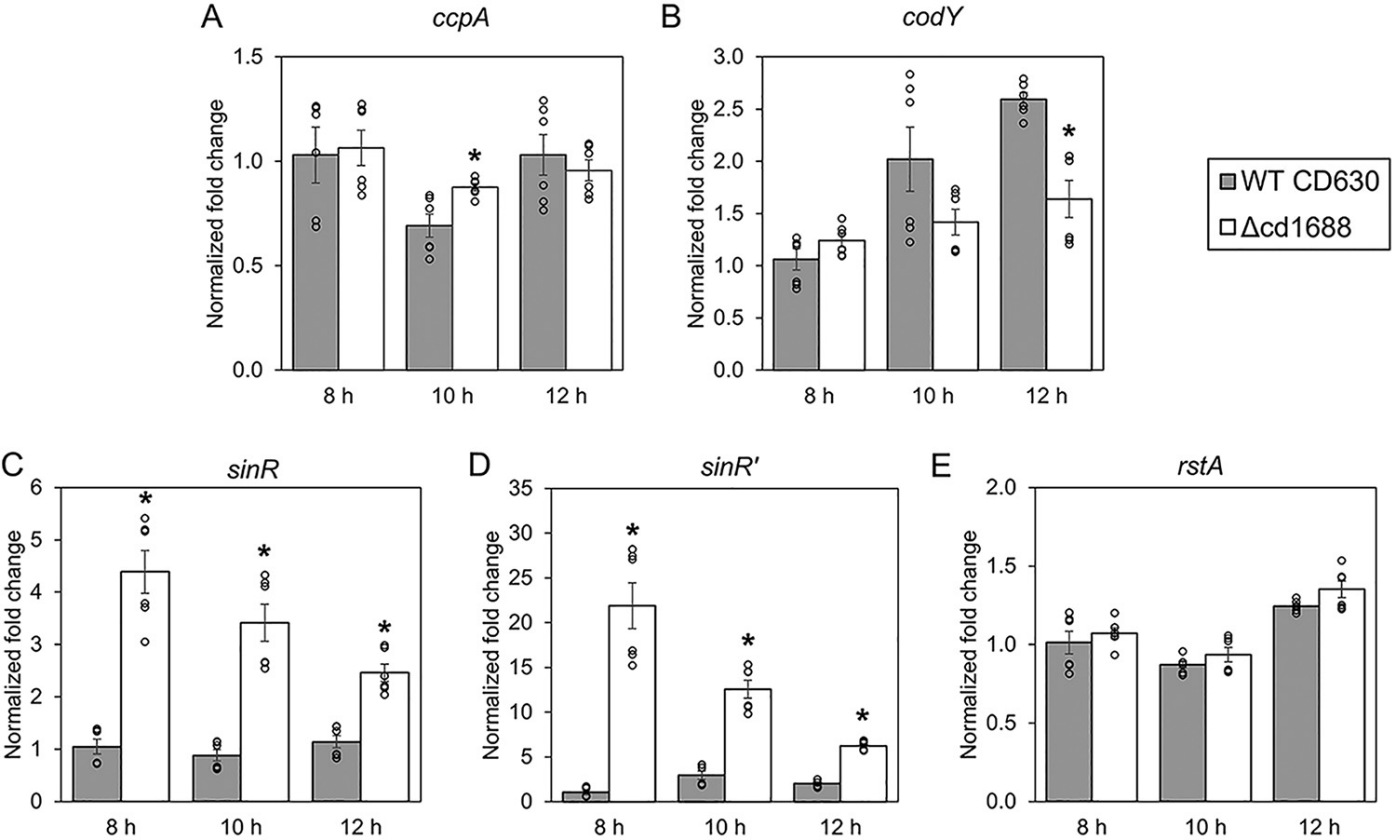
Transcript abundance of some known regulators of sporulation in the C. difficile WT and Δcd1688 strains. Expression of *ccpA* (A), *codY* (B), *sinR* (C), *sinR′* (D), and *rstA* (E) was measured from RNA samples isolated at 8 h, 10 h, and 12 h postinoculation on 70:30 sporulation media from C. difficile WT and Δcd1688 strains. Expression for each gene is presented relative to the WT sample at 8 h. *, *P* ≤ 0.05 as determined by Student's *t* test compared to the C. difficile WT630 strain at the same time point.

C. difficile sporulation is known to be influenced by several global regulators. For example, CcpA (carbon control protein) and CodY (with cofactors BCAAs/GTP) repress sporulation when nutrient levels are high in the environment (23, 24, 37, 38). The *sin* locus encodes *sinR* and *sinR′*, which are inhibitors of sporulation in B. subtilis (39, 40). The mechanism of control of sporulation via SinRR′ in C. difficile remains unclear, but studies have shown that a *sin* locus deletion was asporogenic, SinR positively regulates sporulation, and SinR′ acts as an antagonist through direct binding to SinR, thus negatively influencing sporulation (25, 41). The multifunctional regulator RstA has also been shown to positively affect sporulation through a yet-to-be-identified mechanism (42, 43). To determine if any of these global regulators were differentially expressed in our C. difficile Δcd1688 strain, we measured transcript abundance of these genes during growth on 70:30 sporulation plates via qRT-PCR. For *ccpA*, we only observed a significant difference of expression at 10 h (1.3-fold increase) in the C. difficile Δcd1688 strain (Fig. 4A). Expression of *codY* was significantly different only at 12 h, with a 0.6-fold decrease in the C. difficile Δcd1688 strain (Fig. 4B). In contrast, both *sinR* and *sinR′* transcripts were significantly increased in the C. difficile Δcd1688 strain at all time points tested (Fig. 4C and D). We observed no significant changes in *rstA* expression between the C. difficile Δcd1688 and WT strains at any of the tested time points (Fig. 4E).

### Deletion of *cd1688* results in increased expression of sporulation-specific genes.

**FIG 5:**
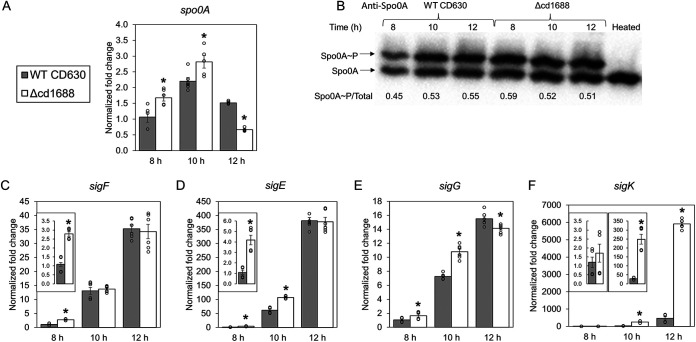
Gene expression profile of sporulation-specific genes and levels of phosphorylated Spo0A in C. difficile WT versus the C. difficile Δcd1688 strain. (A) Transcript abundance of *spo0A* in the Δcd1688 strain at 8 h, 10 h, and 12 h postinoculation on 70:30 medium relative to the WT sample at 8 h. (B) Detection of Spo0A phosphorylation. Cell lysates isolated from C. difficile WT and Δcd1688 strains at 8 h, 10 h, and 12 h postinoculation on 70:30 sporulation agar were resolved via Phos-tag SDS-PAGE and subjected to immunoblotting using anti-Spo0A antibody. (C to F) Transcript abundance of *sigF* (C), *sigE* (D), *sigG* (E), and *sigK* (F). All gene expression was measured via qRT-PCR from RNA samples isolated at 8 h, 10 h, and 12 h postinoculation on 70:30 sporulation media. *, *P* ≤ 0.05 as determined by Student's *t* test compared to the C. difficile WT630 strain at the same time point.

To further investigate how the deletion of *cd1688* increased sporulation efficiency, we examined the C. difficile Δcd1688 strain for differences in the expression of a set of genes known to be involved in initiation of sporulation. The transcript levels of *spo0A*, the master regulator of sporulation, were increased in the C. difficile Δcd1688 strain compared to the WT strain at 8 h (1.7-fold) and 10 h (1.3-fold) after inoculation on 70:30 sporulation agar plates but decreased compared to WT levels at 12 h (0.4-fold) (Fig. 5A). Spo0A-mediated regulation is dependent on phosphorylation and does not necessarily correlate to transcript levels (10, 44, 45). Therefore, we also measured the amount of phosphorylated Spo0A (Spo0A~P) using Phos-tag SDS-PAGE analysis from total protein harvested from the C. difficile Δcd1688 and WT strains at 8 h, 10 h, and 12 h postinoculation on 70:30 sporulation plates. Both unphosphorylated Spo0A and Spo0A~P forms were detected via Western blot analysis with anti-Spo0A antibody (Fig. 5B). We confirmed that the upper band corresponded to Spo0A~P, as upon heating, this band disappeared. At the 8-h time point, the ratio of Spo0A~P to Spo0A was higher in the C. difficile Δcd1688 strain than the WT strain. There was no difference between the strains at 10 h and by the 12-h time point, the ratio of Spo0A~P to Spo0A had decreased in the C. difficile Δcd1688 strain compared to the WT strain.

Two well-established targets of Spo0A~P are the cell type-specific sporulation sigma factors *sigE* and *sigF*, which control transcription of sporulation genes in the mother cell and the forespore, respectively (16). Therefore, we next measured the transcript abundance of these two genes during growth on 70:30 sporulation medium. As shown in Fig. 5C and D, *sigF* transcripts were significantly increased at 8 h (2.8-fold; see inset in Fig. 5C), while the *sigE* transcripts were significantly increased both at 8 h (4.2-fold; see inset in Fig. 5D) and 10 h (1.8-fold) in the C. difficile Δcd1688 strain compared to the WT strain at the same time point. The expression of genes involved in the later stages of sporulation is controlled by SigG and SigK in the forespore and mother cell, respectively. Therefore, we also measured the expression of these genes during growth on 70:30 sporulation plates. In the C. difficile Δcd1688 strain, *sigG* was significantly increased compared to the WT strain at the same time point for both 8-h (1.7-fold) and 10-h (1.5-fold) samples (Fig. 5E) while *sigK* was significantly increased at both 10 h (9.0-fold) and 12 h (14.5-fold) postinoculation (Fig. 5F).

### CD1688 directly binds to the *spoIIR* gene promoter.

**FIG 6:**
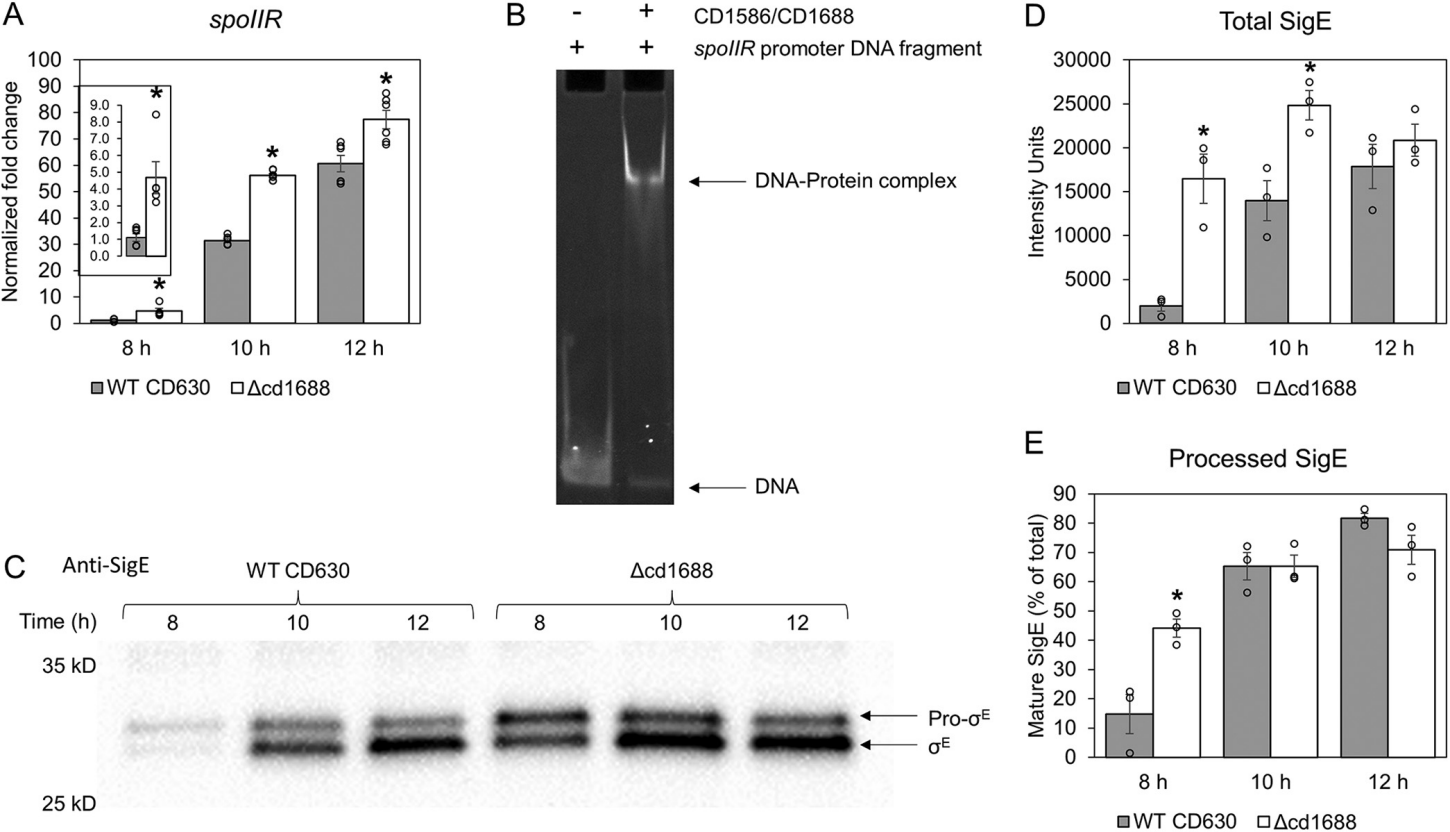
Altered expression of *spoIIR* and SpoIIR-dependent processing of SigE in C. difficile Δcd1688. (A) Transcript abundance of s*poIIR* in the C. difficile Δcd1688 strain at 8 h, 10 h, and 12 h postinoculation on 70:30 sporulation media measured via qRT-PCR relative to the WT strain 8 h sample. *, *P* ≤ 0.05. (B) EMSA analysis indicating *in vitro* binding between RR CD1586/CD1688 and the *spoIIR* promoter region. (C) Western blot analysis of cell lysates isolated from the C. difficile WT and Δcd1688 strains at 8 h, 10 h, and 12 h postinoculation on 70:30 sporulation agar. A total of 15 μg of protein was resolved by SDS-PAGE and subjected to immunoblotting using anti-SigE antibody. (D) The amount of total SigE (pro- and mature forms) was determined by quantification of band intensities in the immunoblot using ImageJ. (E) Percentage of processed, mature SigE. The intensity of mature SigE was divided by total intensity of SigE (pro-SigE plus mature SigE) in each sample.

Of the previously predicted gene targets of CD1688, only *spoIIR* has an identified role in regulating the sporulation pathway in C. difficile (46). During the spore developmental pathway, the mother cell and forespore follow distinct transcriptional programs controlled by the aforementioned cell-specific sigma factors (SigF and SigG in the forespore and SigE and SigK in the mother cell) (16). Pro-SigE must be processed into active SigE in order for the sporulation pathway to proceed in the mother cell (47, 48). Expression of *spoIIR* occurs in the forespore and is mediated by both SigF and Spo0A~P in C. difficile (48). SpoIIR is secreted across the forespore inner membrane space where it interacts with SpoIIGA to initiate cleavage of pro-SigE into mature SigE (19, 49, 50). A previous study in C. difficile confirmed that no processing of pro-SigE occurred in a *spoIIR* deletion strain and cells were arrested at the asymmetric division stage (48). We had observed an increase in expression of *spoIIR* (*cd3564*) during growth in BHIS in the C. difficile Δcd1688 strain (Fig. 2). Thus, we also tested expression of *spoIIR* during growth on 70:30 sporulation plates. Expression of *spoIIR* was increased 4.7-fold, 1.8-fold, and 1.3-fold in the C. difficile Δcd1688 strain at 8 h,10 h, and 12 h, respectively, postinoculation on 70:30 sporulation media (Fig. 6A).

Since *spoIIR* was predicted to contain a CD1688 binding site in the upstream region, we aimed to determine if this RR directly binds to the promoter region of *spoIIR* or if the changes were only due to the observed changes in *sigF* and *spo0A* expression. We performed electrophoretic mobility shift assays (EMSAs) between purified CD1586 (100% identical homolog of CD1688 in strain CDR20291, produced and purified for our previous study [31]) and the *spoIIR* promoter region. CD1586 was shown to cause a gel shift when incubated with the *spoIIR* promoter region, confirming a direct interaction *in vitro* (Fig. 6B and Fig. S6A). No gel shift was observed between CD1586/1688 and a negative-control oligonucleotide, which contained a permuted binding motif (Fig. S6B).

### Deletion of *cd1688* results in increased processing of SigE at an earlier time point.

Based on the previous results and the dependence of SigE processing on SpoIIR (48), we hypothesized that the increased expression of *spoIIR* may lead to a greater amount of mature SigE in the C. difficile Δcd1688 strain than the WT. We performed Western blot analysis on cell lysates at 8 h, 10 h, and 12 h postinoculation on 70:30 sporulation plates using an anti-SigE antibody. Significantly higher total amounts of SigE were observed at the 8-h and 10-h time points in the C. difficile Δcd1688 strain than the WT strain (Fig. 6C and D), which was consistent with the gene expression data (Fig. 5D). Additionally, at 8 h, there was a higher ratio of mature SigE to pro-SigE in the C. difficile Δcd1688 strain than the WT strain (Fig. 6C and E).

Given that the sporulation pathway involves a hierarchical cascade of regulatory events, it is difficult to differentiate the effect of the increase in expression of *spo0A* versus *spoIIR* in the Δcd1688 strain. Spo0A controls the expression of *sigE* and *sigF*. In turn, it is predicted that both Spo0A and SigF control the expression of *spoIIR.* Therefore, we also checked the protein levels of Spo0A and SigF via Western blot analysis at the earliest time point (8 h). SigF was significantly increased in the Δcd1688 strain compared to WT (Fig. S7A). Spo0A was also slightly increased in the Δcd1688 strain, but this change was not statistically significant (Fig. S7B).

### Phosphorylation of CD1688 is necessary for the repression of sporulation in C. difficile.

Typically, phosphotransfer in TCSs occurs between a histidine residue on the HK and an aspartate residue on the RR, and phosphorylation of the RR changes its activity (27). To determine the role of phosphorylation on the activity of CD1688, we constructed a second complement strain harboring a vector with a xylose-inducible promoter driving the expression of *cd1688* containing a single amino acid change of the active site residue from an aspartate (D50) to an alanine (C. difficile Δcd1688::p1688^D50A^), which results in a protein that is unable to be phosphorylated. To evaluate if the hypersporulation phenotype was dependent on the phosphorylation state of CD1688, we compared the sporulation efficiency of the C. difficile Δcd1688::p1688^D50A^ complement strain to C. difficile Δcd1688, Δcd1688::p1688, and WT strains. The C. difficile Δcd1688::1688^D50A^ complement strain had a similar sporulation efficiency to the C. difficile Δcd1688 strain (Fig. 3D). These spores were viable at levels similar to C. difficile Δcd1688 as measured by ethanol resistance assays (Fig. 3D and Fig. S3). This demonstrated that the restoration to WT sporulation efficiency observed for the C. difficile Δcd1688::p1688 complement strain was dependent on phosphorylation of CD1688.

In order to determine if the binding affinity changed between a nonphosphorylatable CD1586/CD1688 and the *spoIIR* promoter, we performed an EMSA with purified CD1586^D50G^, which had been constructed in our previous work (31). We observed the same gel shift pattern that we had with the WT CD1586 (Fig. S6C), indicating that binding can occur when the RR is not phosphorylated.

## DISCUSSION

The ability to produce spores is an essential component of C. difficile transmissibility and persistence. The sporulation pathway has been extensively studied in B. subtilis (18, 51), and although the overall developmental steps are generally conserved in C. difficile, several key regulatory proteins necessary for sporulation initiation are missing in C. difficile (10, 17). Previous studies have established a strong link between certain environmental conditions, including pH and nutrient levels, and sporulation efficiency (23, 24, 26, 37, 52, 53). However, the exact regulatory events that lead to sporulation initiation in C. difficile remain unknown. Here, we determined the cellular role of CD1688, a response regulator that had been previously implicated in a transposon mutagenesis study to be important in sporulation (32). Taken together, our data demonstrated that CD1688 is a novel negative regulator of sporulation in C. difficile.

Previous work in our lab identified the DNA binding motif of RR CD1586/CD1688 and numerous putative gene targets, including several involved in ABC/ion transport, proteolysis, and sporulation (31). Upon deletion of *cd1688*, we observed a significant increase in the majority of these targets, indicating that CD1688 negatively regulates their expression. Furthermore, sporulation efficiency assays demonstrated that upon deletion of *cd1688*, a more than 3-fold increase in sporulation was observed. Sporulation was restored to WT levels when *cd1688* was complemented back into the cells. However, complementation with a nonphosphorylatable CD1688 failed to restore WT sporulation levels, confirming phosphorylation is necessary for repression of sporulation via CD1688. It does not appear that CD1688 acts as a global regulator since the C. difficile Δcd1688 strain exhibited no changes in toxin expression or motility. Moreover, a previous study indicated that the loss of *cd1688* also did not affect the ability of C. difficile to produce biofilms (54). To note, the Dembek et al. study (32), which initially predicted a role of CD1586 in R20291 sporulation, found that an insertion in this gene resulted in a decrease in spore formation, the opposite effect that we found. Given the high number of genes identified to be associated with sporulation (798) in that study, this may be due to a high degree of variability related to the spore purification methods used in the study that likely resulted in many false positives.

The initiation of sporulation in all characterized spore-forming bacteria is dependent on phosphorylation of the master regulator Spo0A (55, 56). Several global transcriptional regulators are known to modulate the expression of *spo0A* and, thus, entry into sporulation. Both CcpA and CodY negatively influence the expression of *spo0A* in response to carbon availability and BCAAs/GTP, respectively (24, 37, 38). One mechanism by which CcpA and CodY affect sporulation is through the direct regulation of *sinRR′* expression (25). SinRR′ has been shown to influence sporulation, toxin production, and motility in C. difficile (41). However, the exact mechanism of this regulation remains unclear. In a previous study, the overexpression of *sinR* resulted in an increase of sporulation, while the overexpression of *sinR′* had the opposite effect (25), further demonstrating the antagonistic relationship between these two proteins. Interestingly, overexpression of the entire *sinRR′* operon also led to an increase in sporulation (25). In our C. difficile Δcd1688 strain, we only observed minor changes in the expression of *ccpA* or *codY*. However, we observed a significant increase in the transcript abundance of *sinR* and, to an even greater extent, of *sinR′* in the C. difficile Δcd1688 strain compared to WT when grown on sporulation plates. We suspect this change in expression was not a direct gene regulation due to the absence of a predicted CD1688 binding motif in the promoter region of *sinRR′*. However, the hypersporulation phenotype observed for C. difficile Δcd1688 may, at least in part, be due to the increase in *sinRR′*. Further work will be necessary to fully understand the relationship between CD1688 and the expression of the *sin* locus.

It is evident that the expression of several of the genes that encode sporulation-specific regulators was increased in the absence of *cd1688*. We observed a modest increase in the expression of *spo0A* at 8 h (1.7-fold) and 10 h (1.3-fold) postinoculation on sporulation media in the C. difficile Δcd1688 strain. However, by the 12-h time point, transcript levels had decreased to below WT levels. We also observed an increase in Spo0A~P at 8 h in the C. difficile Δcd1688 strain, which corresponded to an increase in the expression of the Spo0A~P-dependent targets *sigF* (at 8 h) and *sigE* (at 8 h and 10 h). Overall, the genes necessary for sporulation appear to be expressed earlier in the C. difficile Δcd1688 strain compared to the WT strain.

The activity of SpoIIR is essential in the sporulation process of C. difficile given that a *spoIIR* knockout strain failed to proceed beyond asymmetric division (48). The expression of *spoIIR* in B. subtilis is dependent on SigF and ensures the sequential and temporal expression of the distinct transcriptional programs that occur in the mother cell and forespore (57, 58). Once SpoIIR is produced in the forespore, it is secreted into the inner membrane space, where it signals SpoIIGA to cleave pro-SigE into active SigE in the mother cell (49, 50). This relationship is not as tightly coupled in C. difficile, given that *spoIIR* expression can occur independently of SigF, likely through Spo0A~P (48). However, SpoIIR is still required for pro-SigE processing and, thus, the continuation of transcription of sporulation genes in the mother cell. The promoter region upstream of *spoIIR* was predicted to contain a CD1688 binding motif (31). The expression of *spoIIR* was increased in the C. difficile Δcd1688 strain at all time points measured postinoculation on 70:30 sporulation plates, suggesting that CD1688 typically negatively regulates *spoIIR*. We further confirmed that RR CD1586/CD1688 has the ability to directly bind to the *spoIIR* promoter via EMSA analysis. We hypothesized that the increase in *spoIIR* expression in the C. difficile Δcd1688 strain could lead to an increase in pro-SigE processing in the Δcd1688 strain compared to WT, which was confirmed via Western blot analysis. The total amount of SigE protein was higher in the C. difficile Δcd1688 strain during growth on sporulation media, and the ratio of mature SigE to pro-SigE was also significantly higher at the 8-h time point. We considered that a possible mechanism contributing to the hypersporulation phenotype of the C. difficile Δcd1688 strain could be through this derepression of *spoIIR* in our mutant. We did not observe any difference in binding between a nonphosphorylatable mutant of CD1586/1688 and the *spoIIR* promoter compared to the WT protein *in vitro*. Even though the binding was not affected, we have not yet determined how the cellular activity of CD1688 changes upon phosphorylation. The derepression we observed for *spoIIR* in the Δcd1688 strain could be contributing to the speed in which the sporulation pathway progresses in this mutant. Our future work will focus on deciphering the individual contributions of the increased expression of *spo0A* versus *spoIIR* to the hypersporulation phenotype of the Δcd1688 strain.

**FIG 7:**
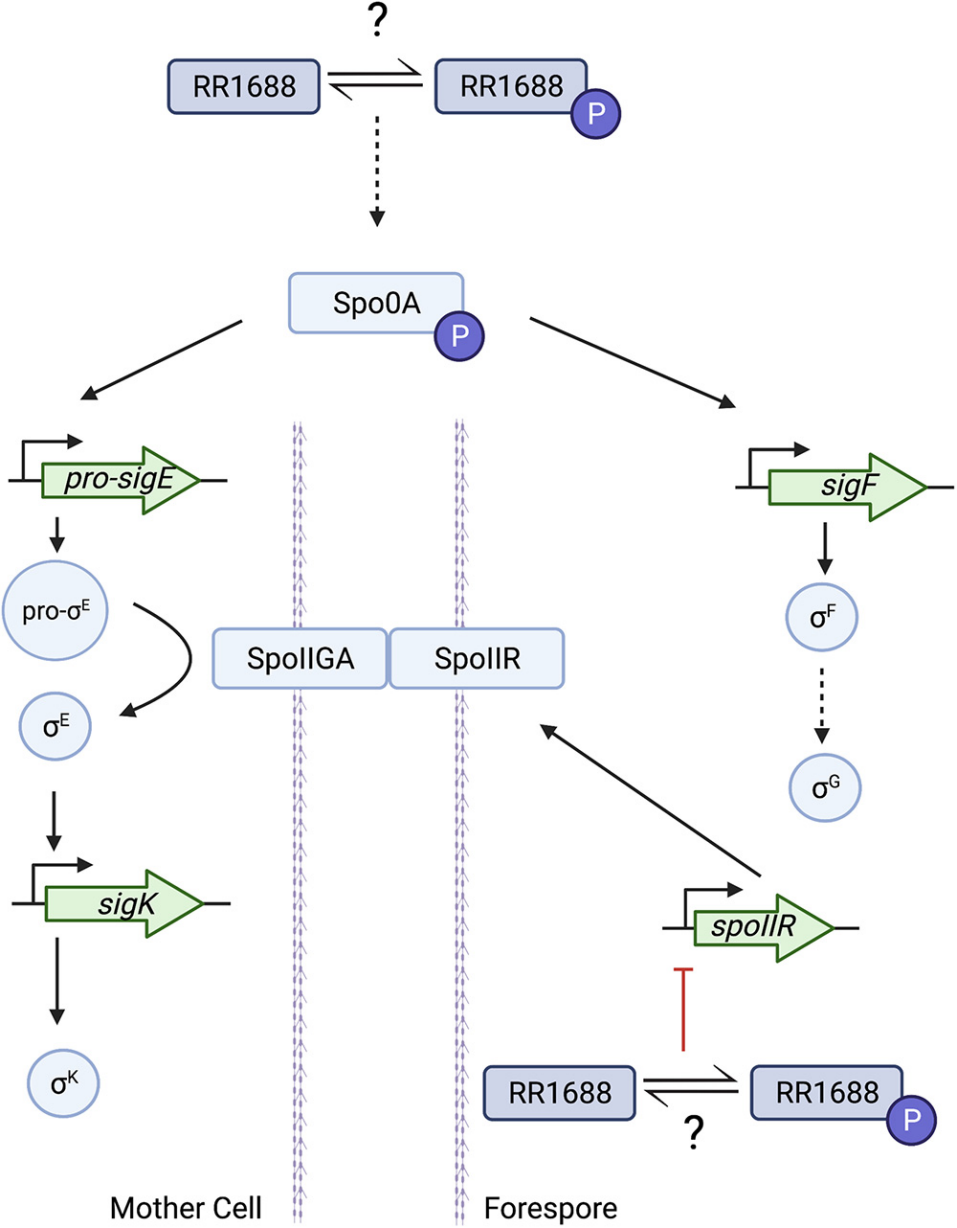
Working model of regulation of sporulation by CD1688. Green arrows represent genes, blue rectangles or circles represent proteins, solid lines indicate known interactions, dashed lines indicate indirect regulation, and red line indicates our proposed regulation via CD1688. Sporulation genes are grouped by their cellular location (either mother cell or forespore). We do not have any evidence that CD1688 is specific to any cellular compartment. Purple circle represents protein phosphorylation. Figure was created using BioRender.com.

In summary, our study shows that CD1688 plays an important regulatory role in the sporulation pathway in C. difficile. CD1688 is the first-described transcriptional regulator that directly binds to the promoter region of *spoIIR* and may represent an additional checkpoint in the sporulation pathway. Collectively, our findings suggest that a yet-to-be-determined environmental signal activates the TCS via autophosphorylation of HK CD1689, followed by subsequent phosphoryl transfer to CD1688, which results in repression of sporulation (Fig. 7). Given that several of the other predicted targets of CD1688 encode ABC and ion transporters, future work will focus on determining if there is a nutritional link to this regulation, similar to what has been observed for several other sporulation regulators in C. difficile (24, 37, 38). Future studies evaluating the role of the other predicted CD1688 gene targets in sporulation, the effect of phosphorylation on its ability to bind certain promoters, and the identification of the external stimuli sensed by the HK CD1689 will also add to our mechanistic understanding of spore development in C. difficile and will perhaps reveal new gene targets for therapeutic development.

## MATERIALS AND METHODS

### Bacterial cultivation.

The bacterial strains and plasmids used in this study are listed in Table S1 in the supplemental material. C. difficile 630 strains were routinely cultured in brain heart infusion (Sigma-Aldrich) medium supplemented with yeast extract (BHIS) containing appropriate antibiotics (2-10 μg/mL thiamphenicol, 250 μg/mL d-cycloserine, or 8 μg/mL cefoxitin) or in 70:30 sporulation medium that contained 70% SMC (90 g Bacto peptone, 5 g protease peptone, 1 g ammonium sulfate, 1.5 g Tris base, and 15 g agar per L) and 30% BHIS (59). Taurocholate (TA) was added to a final concentration of 0.1%, where indicated, as a germinant. To induce expression of *cd1688* in the complement strains, 0.1% xylose was added to growth media where indicated. C. difficile was cultured in an anaerobic chamber (Coy Laboratory Products) at 37°C with an atmosphere of 3.5% H_2_ and 5 to 8% CO_2_ and balanced with N_2_ (60). Escherichia coli strains were grown at 37°C in Luria-Bertani media (LB) with antibiotics as necessary (20 μg/mL chloramphenicol or 50 μg/mL kanamycin).

### Strain and plasmid construction.

The CRISPR-Cas9 nickase vector used for generating the mutant strain was constructed using pTMS001 as a backbone as described previously (33). Briefly, three elements were assembled into the backbone, the left homology arm, the right homology arm, and a custom guide RNA (gRNA) designed to target *cd1688*. These regions were PCR amplified from CD630 genomic DNA (gDNA) using Q5 high-fidelity DNA polymerase (NEB) or synthesized by Integrated DNA Technologies (IDT) as a gBlock (oligonucleotides are listed in Table S2). The backbone was linearized via PCR using primers (Pr484 and Pr485) and treated with DpnI following the manufacturer’s protocol (NEB) to remove any remaining plasmid template. All fragments (right and left homology arms, linearized backbone, and gRNA) were assembled using HiFi Assembly master mix (NEB) following the manufacturer’s protocol, generating the deletion vector (pTMS011).

To complement the *cd1688* deletion, the coding sequence of *cd1688* was PCR amplified using primers BAL36F and BAL36R and cloned into SacI/BamHI-digested pAP114, generating pTMS012, which resulted in the expression of *cd1688* being driven by a xylose-inducible promoter. pAP114 was a gift from Craig Ellermeier and David S. Weiss (Addgene plasmid number 120799) (35). The Q5 site-directed mutagenesis kit (NEB) was used with primers BAL38F and BAL38R to modify the complement vector pTMS012 to contain an aspartate-to-alanine mutation at amino acid 50, generating pTMS013. Vectors were transferred into DH5α or NEB 10-β cells via transformation, plated on LB with chloramphenicol, and sequence verified (Oklahoma Medical Research Foundation).

### Conjugation.

Plasmids were transferred into the conjugal donor strain E. coli CA434 via electroporation and then into CD630 by conjugation as previously described (61). To generate the C. difficile Δcd1688 mutant, several clones were selected and further transferred 3 to 5 times sequentially in BHIS with thiamphenicol. Clones were screened via PCR to confirm loss of *cd1688*, and Sanger sequencing further confirmed the desired mutation was present. Strains were passaged several times in nonselective BHIS to cure the plasmid.

### RNA extraction, cDNA synthesis, and qRT-PCR.

Overnight C. difficile cultures were diluted in fresh BHIS medium to an optical density at 600 nm (OD_600_) of 0.05 and grown to either exponential (OD_600_, ~0.5) or stationary growth phase (OD_600_, ~1.0). Cell pellets were resuspended in TRIzol (Thermo Fisher). Cells grown on 70:30 sporulation agar were scraped from plates at the specified time points, washed with phosphate-buffered saline (PBS), and resuspended in TRIzol. RNA was extracted using the Direct-zol RNA MiniPrep Plus kit following the manufacturer’s protocol (Zymo Research). Samples were treated with Turbo DNase I to remove contaminating gDNA, following the manufacturer’s protocol (Thermo Fisher). cDNA was synthesized from 1 μg of total RNA using LunaScript RT supermix (NEB). Samples containing no reverse transcriptase enzyme were used as the template in subsequent qPCRs to ensure no gDNA contamination was present. qRT-PCR analysis was performed in technical triplicate with 25 ng cDNA per reaction mixture using LunaScript qPCR master mix (NEB). Samples were normalized to the housekeeping gene *rpoC* and/or *rpoB*, and differences in gene expression were calculated using the Pfaffl comparative method (62). The normalized fold change was calculated for expression of each gene in the Δcd1688 strain relative to expression in the WT630 strain in the same growth conditions or at the 8-h time point as indicated in the figure legends. Efficiencies of the primers were analyzed using serial dilutions of cDNA. Primers are listed in Table S2. We performed three technical replicates for three biological replicates and presented the mean along with the standard error of the mean. Statistical significance was calculated using two-tailed Student’s *t* test. All statistical tests were performed in R version 4.1.2 (63).

### Sporulation assays.

Sporulation efficiency determination and microscopy imaging were performed as described previously (34, 52, 64). C. difficile cultures were grown overnight in BHIS-TA and then back diluted in fresh BHIS-TA to an OD_600_ of 0.05. Once the cultures reached an OD_600_ of 0.5, 150 μL was spread on prereduced 70:30 sporulation plates. Cells/spores were harvested at the specified time points. For microscopy (phase contrast), cells were scraped from plates, resuspended in PBS, and removed from the anaerobic chamber. Cells were pelleted and resuspended in 1 mL of PBS. A small volume (5 to 8 μL) of the resuspended culture was applied to a 0.7% agarose pad, covered with a cover slip, and imaged via phase-contrast microscopy using an Olympus BX51 microscope. At least three fields were obtained per strain, and vegetative cells and spores were counted. For ethanol resistance assays, cells were scraped from 70:30 sporulation plates after 24 h growth and resuspended in 5 mL of BHIS to an OD_600_ of 1.0. Serial dilutions were performed and plated onto BHIS agar plates to enumerate vegetative cells. A 0.5-mL aliquot of the cell mixture was mixed with 0.2 mL of distilled water (dH_2_O) and 0.3 mL of 95% ethanol (final concentration, 28.5% ethanol) and incubated for 15 min at room temperature. The sample was then serially diluted and plated on BHIS-TA plates to enumerate ethanol-resistant spores. The percent sporulation efficiency was calculated as $\frac{\text{number}\;\text{of}\;\text{EtOH}\;-\;\text{resistant}\;\text{spores}}{\text{number of vegetative cells}+\text{number}\;\text{of}\;\text{EtOH}\;-\;\text{resistant}\;\text{spores}}\times 100$.

Three biological replicates were performed, and the standard error of the mean was calculated. A one-way analysis of variance (ANOVA) followed by Dunnett’s multiple-comparison test was performed to measure statistical significance compared to the WT strain.

### Western blot analysis.

Cultures of C. difficile were grown overnight in BHIS-TA and then back diluted to an OD_600_ of 0.05 using fresh BHIS-TA and grown to an OD_600_ of 0.5. Aliquots of 150 μL were then spread onto 70:30 sporulation plates, grown for the indicated times at 37°C, and collected as described above. Cell pellets were resuspended in 2% SDS containing 4 M urea and lysed using bead beating. Samples were diluted, and protein concentrations were determined using the Pierce bicinchoninic acid (BCA) protein assay kit (Thermo Fisher).

Total protein samples (15 μg) were resolved on a 12% SDS-PAGE gel and transferred to a nitrocellulose membrane. The membrane was blocked using 1% milk in TBST (Tris-buffered saline with 0.1% Tween 20) and probed with the specified primary antibody (diluted 1:2,000) overnight at 4°C, followed by washing with TBST and incubation with horseradish peroxidase (HRP)-conjugated secondary antibody (diluted 1:3,000; Sigma) for 30 min at room temperature. Primary antibodies were a generous gift from Aimee Shen (Tufts University). The blots were developed using the Bio-Rad Clarity Western ECL kit, and proteins were visualized using a ChemiDoc MP charge-coupled-device (CCD) imaging system. Densitometry was performed using ImageJ. Western blotting was performed for three biological replicates, and a representative blot is presented.

Phos-tag gel Western blotting was performed as described below. Gels were prepared according to the manufacturer’s instructions (Fujifilm Wako Chemicals Inc., USA), and 12% SDS-PAGE gels were copolymerized with 50 μM Phos-tag acrylamide and 10 μM MnCl_2_. Total protein lysates (10 μg) were resolved by electrophoresis and then electroblotted to a nitrocellulose membrane. Western blots were performed as described above except the primary Spo0A antibody was diluted 1:1,000. To confirm that the slower-migrating bands represented phosphorylated proteins, duplicate samples were heated at 95°C for 5 min to hydrolyze the phosphoryl group prior to loading on the gel.

### EMSA.

EMSAs of CD1586 or a nonphosphorylatable mutant (CD1586^D50G^) and DNA were run as previously described (31). Briefly, pairs of single-stranded oligonucleotides (Table S2) were annealed at 95°C for 5 min in 10 mM Tris, pH 8.0, and 50 mM NaCl. Samples were cooled to room temperature. Protein (concentration as indicated) and double-stranded DNA (dsDNA; 0.5 μM) were incubated at room temperature for 10 min in binding buffer (10 mM Tris, pH 8.0, 50 mM NaCl, and 10 mM MgCl_2_). Samples were separated on a prerun 10% polyacrylamide gel and run at 120 V for 1 h, submerged in ice. DNA was stained using 0.5× TBE containing ethidium bromide for 5 min. Images were captured using a Gel Logic 100 system with a UV transilluminator.
