# Supplementary material for: Response Regulator CD1688 Is a Negative Modulator of Sporulation in Clostridioides difficile
Source: J Bacteriol. 2022 Jul 19;204(8):e00130-22. doi: 10.1128/jb.00130-22 (PMC9380558; doi:10.1128/jb.00130-22)
Supplement: Supplemental file 1 — Supplemental material. Download jb.00130-22-s0001.pdf, PDF file, 0.9 MB [file jb.00130-22-s0001.pdf]

Supplementary Materials

Response regulator CD1688 is a negative modulator of sporulation in *Clostridioides difficile*

Megan L. Kempher[1,2], Savannah C. Morris[1], Tyler M. Shadid[2], Smita K. Menon[1], Jimmy D. Ballard[2], Ann H. West[1]

[1]University of Oklahoma, Department of Chemistry and Biochemistry, Norman, OK

[2]University of Oklahoma Health Sciences Center, Department of Microbiology and Immunology,

Supplemental Methods

Motility assay. Cultures of *C. difficile* were grown overnight in BHIS, then back diluted to an OD_{600} of 0.5 using fresh BHIS. Plates for motility were prepared by adding 25 mL of BHIS containing 0.4% agar (w/v) into each plate and let sit until dry (1). Plates were then spot inoculated with 5 µL of culture of each strain and incubated at 37°C for 5 days. The diameter of each spot was measured every 24 h. Four replicates were performed for each strain and the assay was repeated independently three times.

Table S1. List of strains and plasmids used in this study.

Plasmid or strain	Description	Source or reference
***C. difficile* strains**		
CD630	Clinical isolate of *C. difficile*	ATCC
Δcd1688	CD630 with *cd1688* deleted via plasmid pTMS011	This study
Δcd1688::p1688	Δcd1688 with pTMS012	This study
Δcd1688::p1688^{D50A}	Δcd1688 with pTMS013	This study
***E. coli* strains**		
CA434	Conjugal donor strain HB101 carrying R702	Chain Biotech (2)
**Plasmids**		
pMTL84151	*E. coli- C. difficile* shuttle vector	Chain Biotech (2)
pTMS001	Codon-optimized p*fdx-cas9-nickase* in pMTL84151	(3)
pTMS011	*cd1688*-targeted gRNA and customized homology region in pTMS001	This study
pAP114	Plasmid carrying a xylose inducible promoter in front of *mCherry*	(4)
pTMS012	pAP114 with *mCherry* removed and replaced with *cd1688*	This study
pTMS013	pTMS012 with D50A site directed mutation of *cd1688*	This study

Table S2. List of oligos used in this study.

Primer	Sequence (5' to 3')	Use	Source or reference
Pr415	caacgtcgtgactgggaaaaccctggcatgtgaaaagatgaac	To amplify left HDT to flank *cd1688* F	This study
Pr416	tcttatttatcataagtatcccctacctttatttg	To amplify left HDT to flank *cd1688* R	This study
Pr417	taggggatacttatgataaataagaatgtatttaccag	To amplify right HDT to flank *cd1688* F	This study
Pr418	tgttgcaacgagagtttccttcattgcagtag	To amplify right HDT to flank *cd1688* R	This study
Pr484	cgcccgcccttaagtctaaaaattaggggagatg	To linearize pTMS001 F	(3)
Pr485	cagggttttcccagtcacgacgttgtaaaacgac	To linearize pTMS001 R	(3)
Pr419	aatgaaggaaactctcgttgcaacaaattgatgagcaatgcttttttataatgccaactttgtacaaaaaagcaggctccgaattcgcccttggaattctagacataatatattgacaaatttattttttaaagttaaaattaagttggaaactgaaaagtgtgaagagttttagagctagaaatagcaagttaaaataaggctagtccgttatcaacttgaaaaagtggcaccgagtcggtgctttttttatcgatcggctctagacgatcgcgcccgcccttaagtctaaaaatta	Gblock containing custom gRNA for *cd1688* deletion	This study
BAL36F	gaaaacgatagttatgaagtgagctcctgcagtaaaggagaaaattttatgaaggttttaatagttgaagataataag	to amplify *cd1688* to make pTMS012 F	This study
BAL36R	tttaaagttttattaaaacttatagttatttatcatcgaatatatatcctacc	to amplify *cd1688* to make pTMS012 R	This study
BAL39F	gttttacaacgtcgtgactg	pTMS011 insert screening primer F	This study
BAL39R	ccaaatccttacatctcccc	pTMS011 insert screening primer R	This study
BAL37F	cagtaaccaatttgatattcctcct	sequencing primer for pTMS012 F	This study
BAL37R	tggcatctttttatttagggatttc	sequencing primer for pTMS012 R	This study
BAL38F	gtcattttagctttaatgcttcctaatataaatg	Site directed mutagenesis primer to generate D50A mutation in CD1688 F	This study
BAL38R	taaatcatatatattttgatttactaagtataaag	Site directed mutagenesis primer to generate D50A mutation in CD1688 R	This study
Pr445	agatgacaaagtagaagcat	*cd1688* qPCR F	This study
Pr446	gcgtataccctagctact	*cd1688* qPCR R	This study
Pr447	gggacttacatatagtggaattga	*cd1689* qPCR F	This study
Pr448	ggatctttcagtcttattgggt	*cd1689* qPCR R	This study
Pr449	ctagctgctcctatgtctcacatc	*rpoC* qPCR F	(5)
Pr450	ccagtctctcctggatcaacta	*rpoC* qPCR R	(5)
Pr451	aactagggccagaggaaataac	*rpoB* qPCR F	(5)

Pr452	ctgagtctacttctgcacctattc	*rpoB* qPCR R	(5)
Pr453	aagcttatacctggaacagaaa	*cd1685* qPCR F	This study
Pr454	ccttccaatttccttggcttat	*cd1685* qPCR R	This study
Pr459	gcaaacagcaatacagatga	*cd3564* qPCR F	This study
Pr460	ttgaagctttggctgtaaat	*cd3564* qPCR R	This study
BAL30F	ttgagtctcttgaactggtctagg	*spo0A* qPCR F	(6)
BAL30R	ctcaaagcgcaataaatctaggagc	*spo0A* qPCR R	(6)
BAL31F	tgactttacactttcatctgtttctagc	*sigE* qPCR F	(6)
BAL31R	gggcaaatatacttcctcctccat	*sigE* qPCR R	(6)
BAL32F	cgctcctaactagacctaaattgc	*sigF* qPCR F	(6)
BAL32R	ggaagtaactgttgccagagaaga	*sigF* qPCR R	(6)
Pr546	aggcaggtttacatccaacata	*sinR* qPCR F	(7)
Pr547	agtggtatgtctaaagcagtagc	*sinR* qPCR R	(7)
Pr548	aaagacttaaagaagaacggaaaa	*sinR'* qPCR F	(7)
Pr549	ttggattctttttaccactttcg	*sinR'* qPCR R	(7)
BAL23F	tcttgttcaactatccatgaaatcataac	*ccpA* qPCR F	(8)
BAL23R	aaatgggatagaagaggttgctaaa	*ccpA* qPCR R	(8)
BAL22F	ctcatcttctataactgaactgtcttgaac	*codY* qPCR F	(8)
BAL22R	tttgatttactggccggagcattg	*codY* qPCR R	(8)
BAL20F	ttattaaatctgtttctccctcttca	*tcdR* qPCR F	(9)
BAL20R	agcaagaaataactcagtagatgatt	*tcdR* qPCR R	(9)
BAL18F	gcagtcactggatggagaatta	*tcdA* qPCR F	(3)
BAL18R	agatgatagcagtgtcaggattg	*tcdA* qPCR R	(3)
BAL19F	gaaggattacctataattgc	*tcdB* qPCR F	(3)
BAL19R	ctgccattatacctatcttag	*tcdB* qPCR R	(3)
BAL40F	gaaaaacccttaacccctga	*sigK* qPCR F	(10)
BAL40R	tcatcctgatcttccgttga	*sigK* qPCR R	(10)
BAL33F	gtggtgttaatacatcagaacttcc	*sigG* qPCR F	(6)
BAL33R	caaactgttgtctggcttcttc	*sigG* qPCR R	(6)
BAL24F	ttgcaagagttacttcattctgattt	*rstA* qPCR F	(8)
BAL24R	tgtaagatagccttagcttcatcaata	*rstA* qPCR R	(8)
Pr443	atgtatcatttgtagttgcaggac	*cd0552* qPCR F	This Study
Pr444	ctttaattagggcaggaatcagtatc	*cd0552* qPCR R	This Study
Pr455	gggttccttctgccaataaa	*cd3263* qPCR F	This Study
Pr456	ccaacaggtactcctataacaag	*cd3263* qPCR R	This Study
Pr457	tctacgccgatagttgtaaatg	*cd3284* qPCR F	This Study
Pr458	agttcctacaccttgtgtttc	*cd3284* qPCR R	This Study
Pr495	cataagacaagcagaccctagaa	*cd0598* qPCR F	This Study
Pr496	agctgccaactcaccattag	*cd0598* qPCR R	This Study
Pr497	gactccctatcaaaggtggtaaat	*cd0649* qPCR F	This Study
Pr498	cttcttctggcaatccctctac	*cd0649* qPCR R	This Study
Pr499	gtagggcatggagaagaagaaa	*cd0656* qPCR F	This Study
Pr500	gtcctgcactaccaccaaata	*cd0656* qPCR R	This Study
Pr501	tgaagcctttccagcaagtaata	*cd0684* qPCR F	This Study
Pr502	tcctagaggacaagcattaggt	*cd0684* qPCR R	This Study
Pr505	ggaccaagtggatgtggaaa	*cd1024* qPCR F	This Study
Pr506	cttgtctttcatatggtggcaaat	*cd1024* qPCR R	This Study

Pr515	ctagctgtatcagttgggtcaaa	*cd1729* qPCR F	This Study
Pr516	ctcgacgtgcagctgtaat	*cd1729* qPCR R	This Study
Pr519	atcacttcgtatccaggaacttt	*cd2102* qPCR F	This Study
Pr520	ggtaagggaaacaggcgtaata	*cd2102* qPCR R	This Study
Pr527	gtgcataacctttagcatgttgta	*cd2582* qPCR F	This Study
Pr528	aggctgtaaggttgtcagaag	*cd2582* qPCR R	This Study
Pr529	ccatagaggctagcatatccataat	*cd3056* qPCR F	This Study
Pr530	catgaccctctaacagcaagt	*cd3056* qPCR R	This Study
Pr531	aaacatactcgctgcactcg	*cd3416* qPCR F	This Study
Pr532	gccagtgggttcaactaaca	*cd3416* qPCR R	This Study
Pr227	aatattatgaataaattaaatttaat	CDR20291 *spoIIR* EMSA F	This Study
Pr228	attaaatttaatttattcataatatt	CDR20291 *spoIIR* EMSA R	This Study
Pr155	attttacacgaaatgggcacgaagtatac	Negative control DNA for EMSA F	This Study
Pr156	gtatacttcgtgcccatttcgtgtaaaat	Negative control DNA for EMSA R	This Study

Abbreviations: HDT- homology donor template

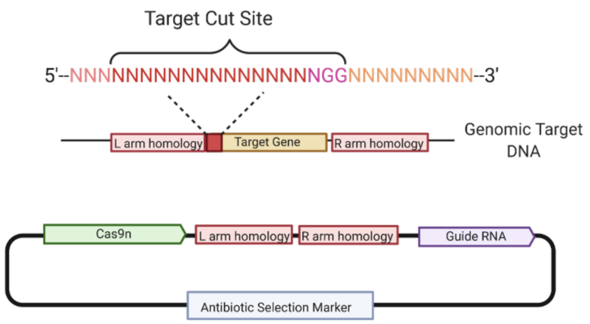

Supplemental Figure 1. CRISPR-Cas9 nickase plasmid design for deletion of *cd1688* in *C. difficile.*

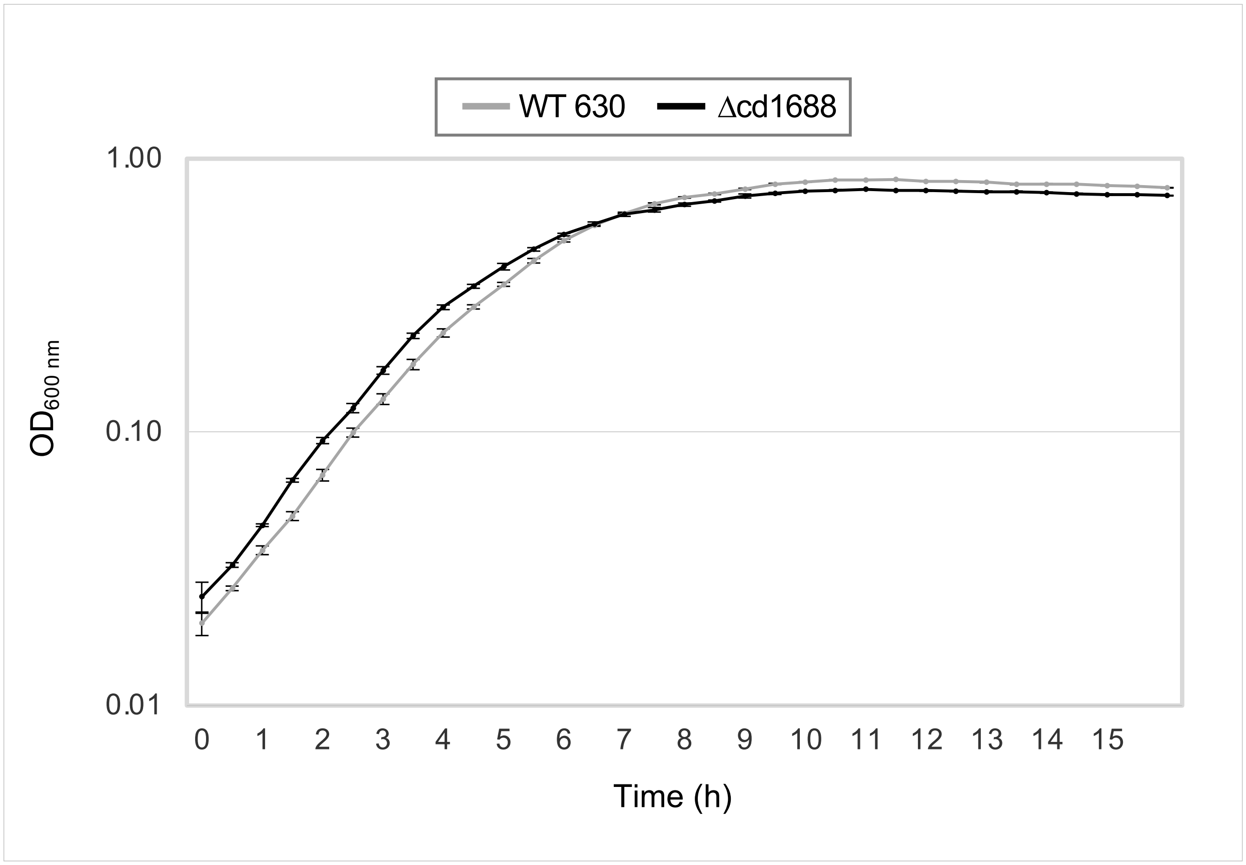

Supplemental Figure 2. Deletion of the *cd1688* gene had no major growth defects. Growth curves of the *C. difficile* wild-type CD630 and Δcd1688 mutant in BHIS media. Shown are the OD_{600} measurements and error bars of three biological replicates.

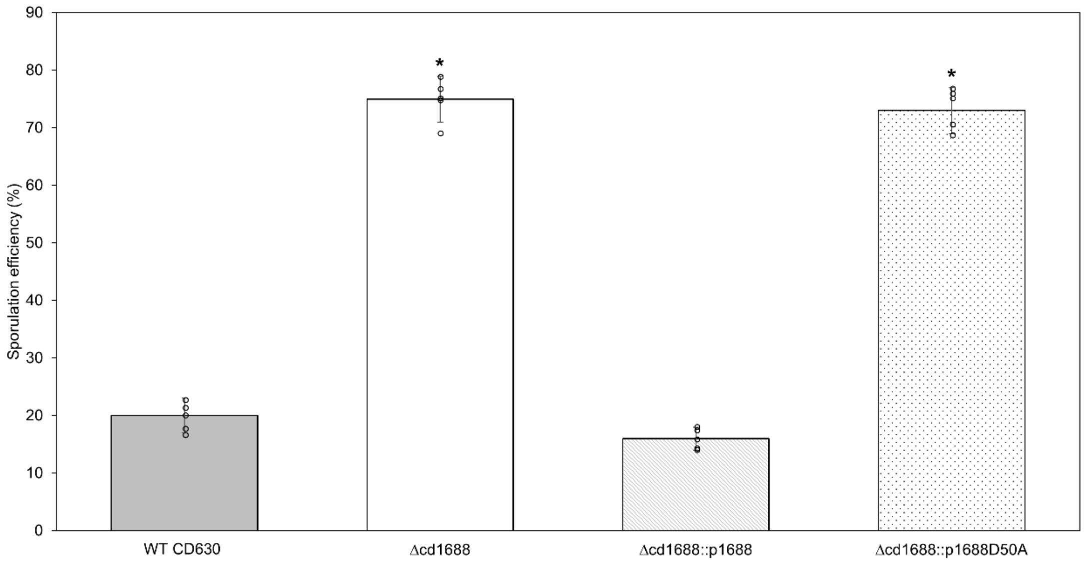

Supplemental Figure 3. Sporulation efficiency of *C. difficile* WT, Δcd1688, Δcd1688::p1688 and Δcd1688::p1688^{D50A} strains grown for 24 h on 70:30 sporulation plates supplemented with 0.1% xylose was calculated as the number of ethanol-resistant spores divided by the total number of cells (spores plus vegetative cells). * Represents $P \leq 0.01$ as determined by a one-way ANOVA followed by Dunnett's multiple comparisons test compared to *C. difficile* WT.

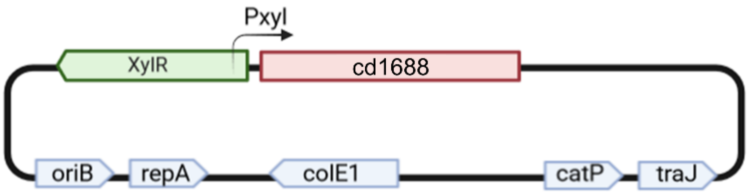

Supplemental Figure 4. Xylose-inducible promoter plasmid design for complementation of *cd1688* in *C. difficile* (4).

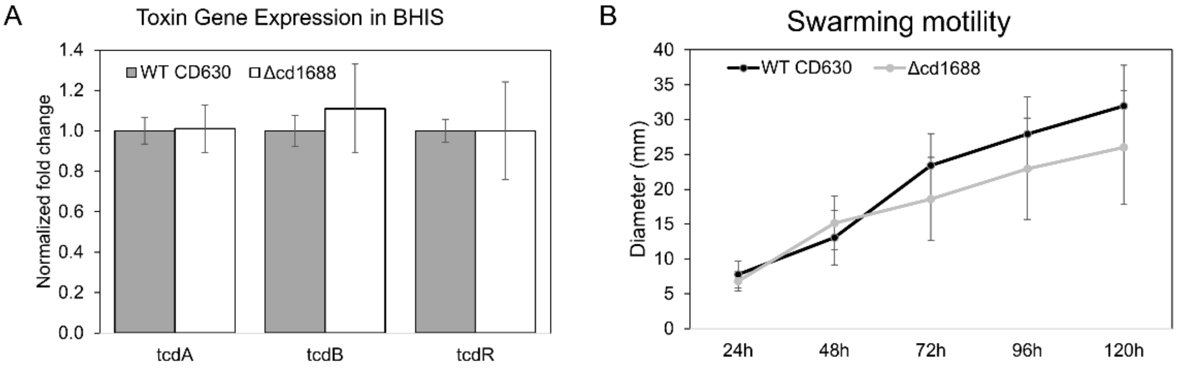

Supplemental Figure 5. Deletion of the *cd1688* gene has no effect on toxin production or motility. A) Transcript abundance of *tcdA, tcdB* and *tcdR* in the *C. difficile* WT and Δcd1688 strains during stationary growth phase in BHIS media. B) Swarming motility as measured by diameter of growth on plates every 24 h for 120 h.

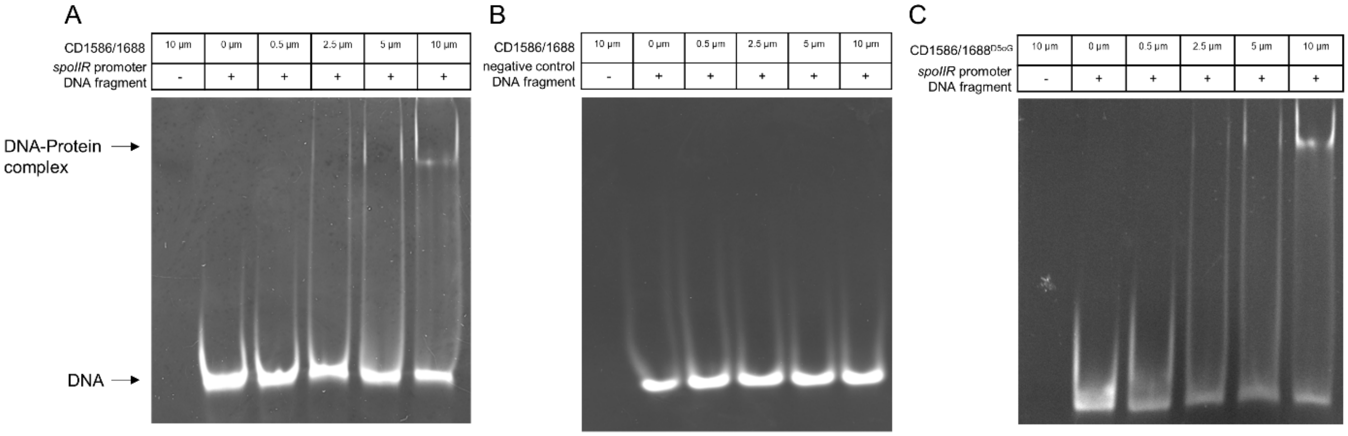

Supplementary Figure 6. EMSA analysis between A) the *spoIIR* promoter region and CD1586/1688, B) negative control DNA and CD1586/CD1688, and C) the *spoIIR* promoter region and a non-phosphorylatable CD1586/CD1688 mutant. The concentration of the DNA oligo is kept constant (0.5 µM) in each reaction.

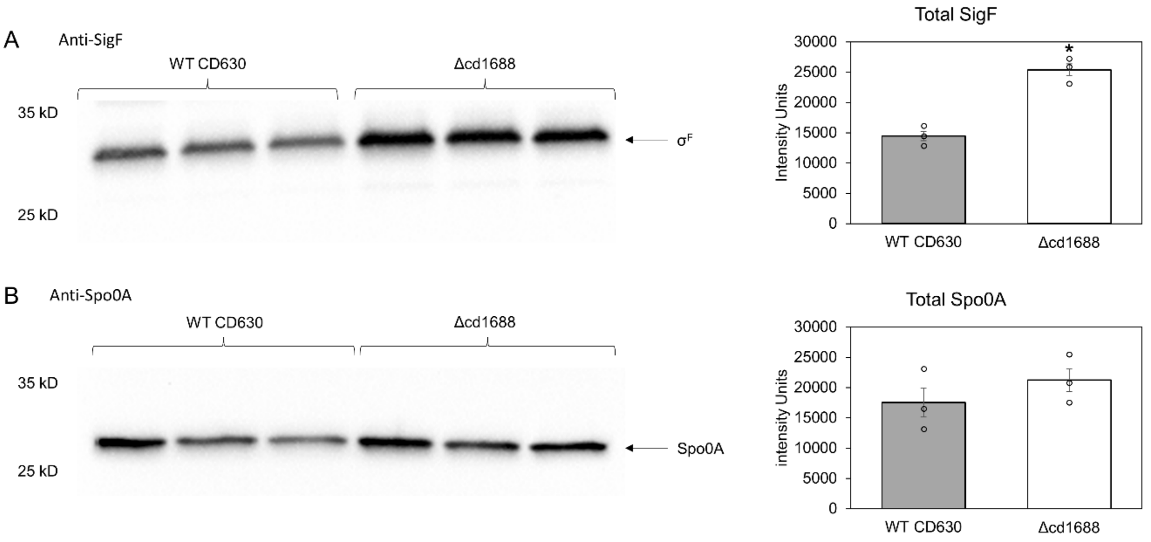

Supplemental Figure 7. Western blot analysis of cell lysates from three biological replicates isolated from the *C. difficile* WT and Δcd1688 strains at 8 h post inoculation on 70:30 sporulation agar. A total of 15 μg of protein was resolved by SDS-PAGE and subjected to immunoblotting using anti-SigF or anti-Spo0A antibodies. * Represents $P \leq 0.05$ as determined by a Student's t-test compared to *C. difficile* WT.

References

1. Baban ST, Kuehne SA, Barketi-Klai A, Cartman ST, Kelly ML, Hardie KR, Kansau I, Collignon A, Minton NP. 2013. The Role of Flagella in *Clostridium difficile* Pathogenesis: Comparison between a Non-Epidemic and an Epidemic Strain. PLoS ONE 8:e73026.
2. Heap JT, Pennington OJ, Cartman ST, Minton NP. 2009. A modular system for *Clostridium shuttle* plasmids. Journal of Microbiological Methods 78:79-85.
3. Ahmed UKB, Shadid TM, Larabee JL, Ballard JD. 2020. Combined and Distinct Roles of Agr Proteins in *Clostridioides difficile* 630 Sporulation, Motility, and Toxin Production. mBio 11:e03190-20.
4. Müh U, Pannullo AG, Weiss DS, Ellermeier CD. 2019. A Xylose-Inducible Expression System and a CRISPR Interference Plasmid for Targeted Knockdown of Gene Expression in *Clostridioides difficile*. Journal of Bacteriology 201.
5. McBride SM, Sonenshein AL. 2011. Identification of a Genetic Locus Responsible for Antimicrobial Peptide Resistance in *Clostridium difficile*. Infection and Immunity 79:167-176.
6. Edwards AN, Nawrocki KL, McBride SM. 2014. Conserved Oligopeptide Permeases Modulate Sporulation Initiation in *Clostridium difficile*. Infection and Immunity 82:4276-4291.
7. Girinathan BP, Ou J, Dupuy B, Govind R. 2018. Pleiotropic roles of *Clostridium difficile* sin locus. PLOS Pathogens 14:e1006940.
8. Edwards AN, Tamayo R, McBride SM. 2016. A novel regulator controls *Clostridium difficile* sporulation, motility and toxin production. Mol Microbiol 100:954-71.
9. McKee RW, Mangalea MR, Purcell EB, Borchardt EK, Tamayo R. 2013. The Second Messenger Cyclic Di-GMP Regulates Clostridium difficile Toxin Production by Controlling Expression of *sigD*. Journal of Bacteriology 195:5174-5185.

10. Saujet L, Shelyakin PV, Gelfand MS, Dupuy B, Henriques AO, Martin-Verstraete I. 2013. Genome-Wide Analysis of Cell Type-Specific Gene Transcription during Spore Formation in Clostridium difficile. PLOS Genetics 9:28.
